# Habitat associations of *Culicoides* species (Diptera: Ceratopogonidae) abundant on a commercial cervid farm in Florida, USA

**DOI:** 10.1186/s13071-019-3626-1

**Published:** 2019-07-26

**Authors:** Dinesh Erram, Erik M. Blosser, Nathan Burkett-Cadena

**Affiliations:** 1 Florida Medical Entomology Laboratory, University of Florida, IFAS, 200 9th St. SE, Vero Beach, FL 32962 USA; 2 Present Address: University of California Davis, One Shields Ave, Davis, CA 95616 USA

**Keywords:** *Culicoides* species, Biting midges, Larval habitat, Environmental variables

## Abstract

**Background:**

Biting midges in the genus *Culicoides* (Diptera: Ceratopogonidae) transmit bluetongue virus (BTV) and epizootic hemorrhagic disease virus (EHDV) to ruminants, thus exerting a significant economic impact on animal agriculture worldwide. However, very little is known about the larval habitat characteristics of *Culicoides* species associated with BTV/EHDV transmission, particularly in southeastern USA, limiting the establishment of effective midge control strategies. In this study, we examined the habitat associations of *Culicoides* species abundant on a commercial cervid farm in Florida, USA and quantified several environmental variables of their habitat to identify the key variables associated with midge abundance.

**Methods:**

Mud/substrate samples from three potential larval habitats on the farm (edges of streams, puddles and seepages) were brought to the laboratory and incubated for adult emergence, and the percentage organic matter, macronutrients, micronutrients, pH, electrical conductivity, moisture and microbial concentrations of the substrate were quantified.

**Results:**

Strong habitat associations were observed for *Culicoides haematopotus* (Malloch) (stream edge), *Culicoides stellifer* (Coquillett) (puddles) and *Culicoides loisae* (Jamnback) (stream edge), the most commonly emerging midge species from the samples. Suspected vector species of BTV/EHDV on the property, *C. stellifer* and *Culicoides venustus* (Hoffman), emerged mainly from habitats with moderate-high levels of pollution (edges of puddles and seepages) as indicated by the relatively higher concentrations/levels of organic matter, nutrients and other environmental variables in these samples. The emergence of *C. insignis* was too low to form any meaningful conclusions. For each *Culicoides* species, only weak positive or negative associations were detected between midge abundance and the various environmental variables quantified.

**Conclusions:**

Habitat associations of *Culicoides* species abundant on a local cervid/animal farm vary, most likely as a function of certain biotic/abiotic characteristics of the habitat. Further studies across a larger spatial and temporal scale will be needed to experimentally evaluate/identify the key factors more strongly associated with the abundance of target *Culicoides* species. This information, in the long term, can be potentially exploited to render local habitats unsuitable for midge oviposition/larval development.

**Electronic supplementary material:**

The online version of this article (10.1186/s13071-019-3626-1) contains supplementary material, which is available to authorized users.

## Background

Biting midges in the genus *Culicoides* (Diptera: Ceratopogonidae) are important from medical and veterinary health perspective worldwide because the blood-feeding nature of females can cause major annoyance, hypersensitivity reactions and/or pathogen transmission to susceptible hosts including humans [1–3]. Among the several pathogen classes *Culicoides* species transmit, two orbiviruses, bluetongue virus (BTV) and epizootic hemorrhagic disease virus (EHDV) (genus *Orbivirus*, family *Reoviridae*) affect ruminants causing significant economic losses in animal agriculture worldwide [4, 5]. Although a variety of domestic and wild ruminants are affected by these viruses, white-tailed deer [WTD; *Odocoileus virginianus* (Zimmermann)] in particular are highly susceptible; therefore, BTV/EHDV exert a significant economic impact on the commercial cervid farming industry in North America [3, 6, 7]. Unfortunately, effective *Culicoides* control strategies do not currently exist, primarily because many of the fundamental biological/ecological aspects of the biting midge species involved in BTV/EHDV transmission are unknown.

In North America, only two biting midge species have been confirmed as vectors of BTV/EHDV to date: *Culicoides sonorensis* (Wirth & Jones) (distributed mainly in western USA) and *Culicoides insignis* (Lutz) (distributed mainly in the extreme southeastern USA) [2, 8–12]. However, other midge species such as *Culicoides debilipalpis* (Lutz), *Culicoides stellifer* (Coquillett), *Culicoides haematopotus* (Malloch), *Culicoides venustus* (Hoffman) and/or others are likely involved in *Orbivirus* transmission, particularly in southeastern USA [11, 13–17].

The immature stages of *Culicoides* species are typically found in different types of semi-aquatic habitats including swamps, marshes, shallow margins of ponds, animal dung pats, pastures, tree holes and others [3]. However, the species involved in animal virus transmission are often abundant near livestock [18]. In North America, much of our knowledge on the larval habitat characteristics of *Culicoides* species associated with *Orbivirus* transmission arises from studies on *C. sonorensis*, the immature stages of which are typically found in animal-waste enhanced muds [19]. In the artificial dairy wastewater ponds of California where the species has been best studied to date, larval densities of *C. sonorensis* have been suggested to be influenced by several environmental factors in the habitat such as manure pollution, pond slope, water level fluctuation, moisture and salinity levels [20–27]. However, very little information is available on the habitat requirements of other suspected/potential vectors of orbiviruses in North America, particularly in southeastern USA, where *C. sonorensis* is rare [14, 16, 17, 28–30]. This represents a significant gap in our understanding of the ecology of *Culicoides* species associated with BTV/EHDV transmission in southeastern USA and remains a major limiting factor in the establishment of effective midge control strategies, particularly in commercial cervid facilities in this region. In this study, we (i) examined habitat associations of different *Culicoides* species on a commercial cervid farm in Florida, USA, and (ii) quantified several environmental variables of the larval habitats to identify the key determinants of midge abundance.

## Methods

### Field site

Our field site was located on a ~ 500-acre private commercial cervid farm [31, 32] in Quincy, Gadsden County, FL, USA, which was affiliated with the University of Florida’s Cervidae Health Research Initiative (CHeRI) programme. The site consisted of vast upland and lowland areas spread across the property, a large artificial pond and a natural slow flowing stream passing through the landscape; soil composition of the county/site being typical sand, silt and clay [33]. The upland areas of the farm were covered mainly with pinopsids such as pine (Class: Pinopsida), while the lowland areas were covered predominantly with a variety of magnoliopsids such as maple, oak, hickory, beech, holly, gum and magnolia (class: Magnoliopsida). The farm consisted of breeding pens for white-tailed deer and an open wooded area (fenced along the borders) that served as a ‘free-ranging preserve’ for white-tailed deer and other cervids [axis deer (*Axis axis*), fallow deer (*Dama dama*), elk (*Cervus canadensis*), sika deer (*Cervus nippon*), deer-elk hybrids (*Cervus nippon* × *Cervus canadensis*) and Pere David’s deer (*Elaphurus davidianus*)] as well as bovid species [blackbuck (*Antilope cervicapra*), gemsbok (*Oryx gazella*), scimitar-horned oryx (*Oryx dammah*), nilgai (*Boselaphus tragocamelus*), water buck (*Kobus ellipsiprymnus*), sheep (*Ovis aries*) and goats (*Capra hircus*)] on the property.

### *Culicoides* larval habitat sample collection

**Fig. 1:**
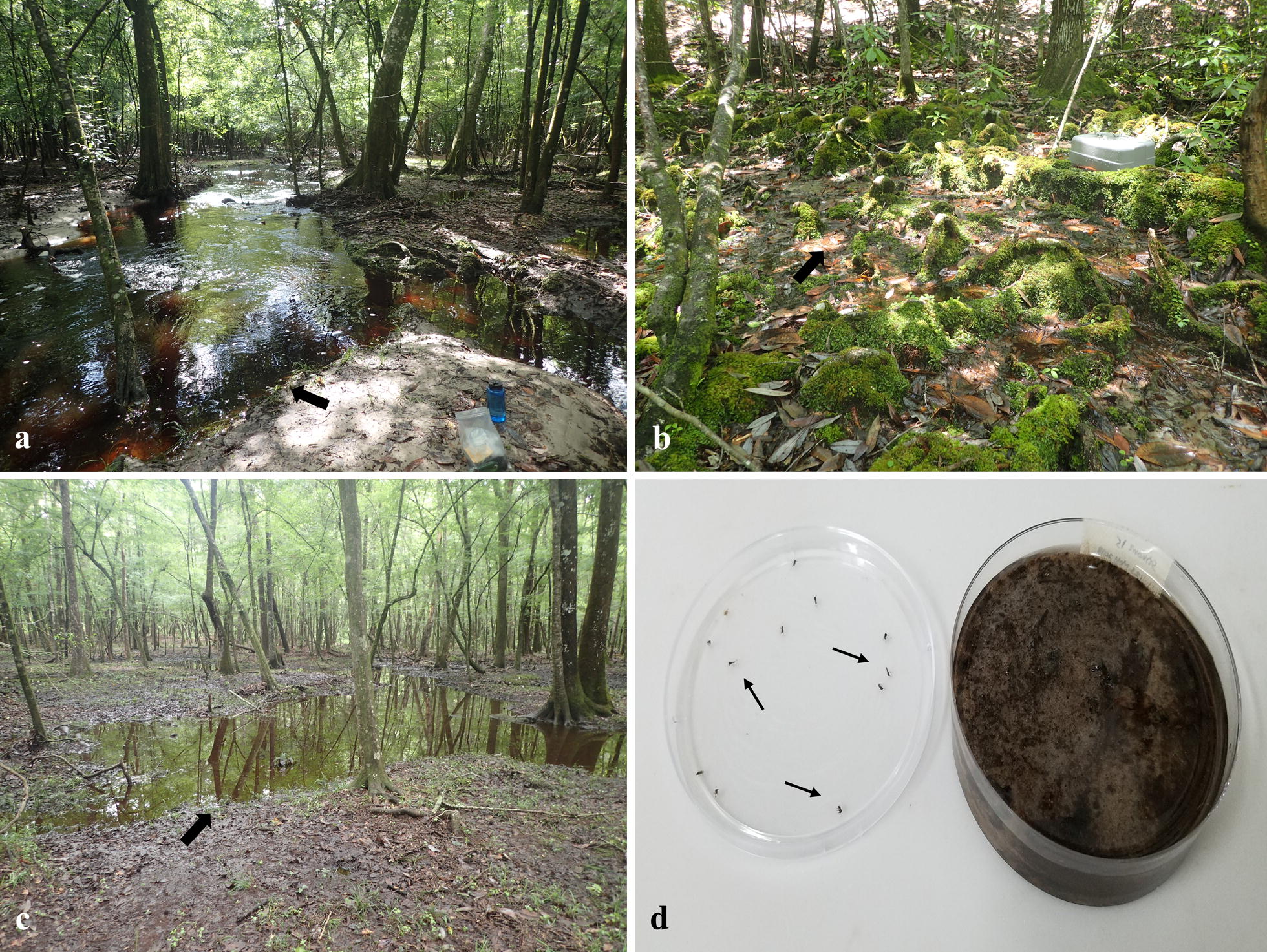
Habitat types from which substrate samples were collected: stream (**a**), seepage (**b**) and puddle (**c**). Arrows in **a**–**c** point to approximate locations from where substrate samples were collected. Substrate samples were brought to the laboratory and incubated in Petri dishes for adult emergence (**d**). Arrows in **d** point to adult midges stuck on the underside of the Petri dish lid, which was smeared with a thin layer of Tanglefoot^®^

Identification of *Culicoides* larval habitats on the farm was based on entomological surveys. Yearly sampling using emergence traps set up at various locations within the farm suggested that the larval habitats of ground-dwelling midge species were present mainly in the lowland areas of the property occupying diverse habitats such as edges of streams, seepages, puddles, ponds and others (unpublished data). Therefore, for the present study, we focused on examining *Culicoides* adult emergence from the three main habitat types that served as larval sites for ground-dwelling midge species on the property: edges of streams, seepages and puddles (Fig. 1a-c). Mud/substrate samples from the three larval developmental sites were collected once a month using a trowel (top few centimeters) from July to September 2017 and adult midge emergence was quantified. Each habitat type had two replicates that were located within 3.0 km of each other within the farm, for a total of six sampling sites per month. From each site, mud/substrate samples were collected from five random areas into five separate Ziploc® bags for a total of 30 samples per month [total number of samples examined = 90 (30 samples × 3 months)]. Random areas were selected by walking to the center of a sampling site and rolling a die to determine the direction and distance walked before sampling. Sampling along stream edges involved walking continuously in one direction with the distance between sampling sites determined by die roll.

### Adult emergence and identification

Mud/substrate samples that were brought to the laboratory were distributed into Petri dishes for adult emergence. Mud/substrate from each of the 30 samples per month [total 90 samples (30 samples × 3 months)] were homogenized gently using a spatula and distributed into five Petri dishes (100 × 25 mm, Fisherbrand, Atlanta, GA, USA; 50.0 ml/dish) for a total of 150 dishes per month [total volume of mud/substrate examined for adult midge emergence = 250.0 ml per sample (50.0 ml mud/substrate × 5 dishes)]. The mud/substrate was sloped gently (~ 15°) in the Petri dishes (Fig. 1d) and tap water was added as needed such that level of standing water in each Petri dish was kept at the pre-determined levels throughout the experiment. The lids of the Petri dishes were smeared with a thin layer of Tanglefoot^®^ (The Tanglefoot Company, Grand Rapids, MI, USA) on the underside to trap insects that emerged for later identification. Overall, the Petri dishes were incubated for two months and all the *Culicoides* adults that emerged during this time were counted, sexed, checked for the presence of nematode parasites in the abdomen [34–36] and identified using morphological keys [28] under a stereoscopic microscope (SMZ 745, Nikon, Melville, NY, USA). Environmental conditions in the laboratory chamber where the Petri dishes were incubated were 26 ± 1 °C, 60–80% RH and a 14:10 h (L:D) photocycle.

### Measurement of environmental variables from habitat samples

A total of 12 different environmental variables were characterized from each sample from each habitat type from each month (from 90 total samples): moisture (%), microbial concentration (CFU/ml), organic matter (%), macronutrients (mg/kg) [phosphorus (P), potassium (K), magnesium (Mg), calcium (Ca)], micronutrients (mg/kg) [copper (Cu), manganese (Mn), zinc (Zn)], pH (pH) and electrical conductivity (dS/m). Moisture levels of the substrate samples were assessed by measuring the wet weight (after excessive standing water was decanted) and dry weight (samples dried in a heating oven for 48 h) of 10.0 ml of the mud/substrate and calculating the difference in percentage [37]. Microbial concentrations (CFU/ml) were enumerated by serially diluting 1.0 ml of mud/substrate in phosphate-buffered saline and spread plating on trypticase soy agar plates, after incubation at 26 °C for 48 h; the microbial concentrations of each substrate were then re-calculated according to its dry weight [38]. In addition, part of the mud/substrate samples were oven-dried and shipped to the University of Florida Soils Laboratory (Gainesville, FL, USA) for the assessment of percentage organic matter, macronutrients (P, K, Mg and Ca), micronutrients (Cu, Mn and Zn), pH and electrical conductivity levels from each sample.

### Statistical analyses

Adult emergence data from the 150 Petri dishes each month were combined into 30 samples per month for statistical analyses. Differences in the total abundance of *Culicoides* adults emerging from different habitat types were analyzed using generalized linear models (GLM) under a negative binomial distribution. Variation in sex ratios of the emerging adults were analyzed using a beta-regression model with logit link function. Differences in the prevalence of parasites in emerging adults were analyzed using GLM with a binomial distribution of the residuals. Variation in the levels of environmental variables measured between different habitat types was examined after log (x + 1) transformation of the data, using linear mixed-effects models with replicate and month nested within habitat type. Means that were significantly different were identified using the Tukey’s *post-hoc* multiple pairwise comparisons test [39].

The importance of environmental variables on the abundance of individual *Culicoides* species within their most productive habitat was assessed using GLM under Poisson distribution [except for *C. venustus* that was analyzed across the two habitats this species emerged from (puddles and seepages, see Results section)]. Variance inflation factors (VIF) were first used to check for collinearities between environmental variables, with a value higher than 10 indicating strong collinearity [40–43]. More specifically, VIFs were determined for environmental variables within stream site, puddle site, and across puddle and seepage habitats [because each emerging *Culicoides* species showed specific associations for these habitat types (see Results section)]. Subsequently, the variable exhibiting the highest VIF value was removed after which VIFs were determined again. This procedure was repeated until all remaining environmental variables in the model showed a VIF value of < 10 (Additional file 1: Tables S1–S3). Copper levels were essentially zero in the stream habitat; therefore, this variable was not included in the VIF analyses within stream habitat (Additional file 1: Table S1) but was retained for VIF analyses within the puddle habitat (Additional file 1: Table S2) and across puddle and seepage habitats (Additional file 1: Table S3). The final full model consisted of 8 environmental variables (scaled) for assessing the abundance of *C. haematopotus* and *C. loisae* (Additional file 1: Table S1) and 11 environmental variables (scaled) for assessing the abundance of *C. stellifer* (Additional file 1: Table S2) and *C. venustus* (Additional file 1: Table S3). Using information-theoretic approach, a set of candidate models were first generated from each of the full models (delta AICc < 2). The relative importance (sum of AIC weights of the variable across all models in the set) and magnitude of the effect of environmental variables in the abundance of individual *Culicoides* species within their most productive habitat(s) was then determined by averaging the selected candidate models [44–46]. Model averaging was considered more appropriate for this study because (i) there is inherent uncertainty in the selection of models when inference is based on only one model that has the highest rank (lowest AIC value) such as in forwards, backwards, or bi-directional model stepwise-selection procedures [46, 47]; (ii) models with ranks of < 2 are the most parsimonious; (iii) weight of the best fit model and the subsequent models adding up to ≥ 0.95 can be used as an equivalent of a 95% certainty [45]; and (iv) the present study is primarily exploratory with virtually nothing being known about the habitat requirements of *Culicoides* species associated with BTV/EHDV transmission, particularly in southeastern USA. All statistical analyses were conducted using R statistical software v.3.30-3 [48] with the packages *Mass* [49], *car* [50], *lsmeans* [51] or *MuMIn* [52] (α = 0.05).

## Results

### *Culicoides* emergence and habitat

A total of 542 *Culicoides* adults emerged from the samples (Table 1). The edges of streams (59.8%, 324/542) and puddles (35.1%, 190/542) were the most productive midge habitats producing almost 95% of the total midges; seepage sites produced only a few (5.2%, 28/542) (LR $\chi^{ 2}_{\left( 2\right)}$ = 52.5, *P* < 0.0001) (Table 1). The overall *Culicoides* diversity in the samples comprised of only five species. *Culicoides haematopotus* was the most abundant species (64.8%, 351/542), followed by *C. stellifer* (21.4%, 116/542), *C. loisae* (8.7%, 47/542), *C. venustus* (4.8%, 26/542) and *C. insignis* (0.4%, 2/542) (Table 1). The numbers of *C. insignis* emerged were too low (*n* = 2 females); therefore, this species was not included in further statistical analyses.

**Table 1:** Total emergence of adult *Culicoides* species from the three habitat types

*Culicoides* spp.^a^	Stream		Seepage		Puddle		Total
	Male	Female	Male	Female	Male	Female	
*C. haematopotus*^b^	97	183	0	2	25	44	351
*C. stellifer*^c^	0	10	6	6	50	44	116
*C. loisae*^b^	15	18	2	5	6	1	47
*C. venustus*^d^	0	0	4	2	12	8	26
*C. insignis*^e^	0	1	0	1	0	0	2
Total	112	212	12	16	93	97	542

^a^Sex-ratios of the emerged adults were not significantly different between *Culicoides* species or habitat types (*P* > 0.05)

^b^The emergence of *C. haematopotus* and *C. loisae* was significantly higher from stream sites than from the other two habitat types (*P* < 0.05)

^c^The emergence of *C.stellifer* was significantly higher from puddle sites than from the other two habitat types (*P* < 0.05)

^d^The emergence of *C. venustus* was not significantly different between puddle and seepage sites (*P* > 0.05)

^e^The emergence of *C. insignis* was too low to be included in statistical analyses

The most abundant species emerging from the stream edge habitats was *C. haematopotus* comprising 86.4% of the total *Culicoides* species emergence from this habitat type (280/324) (Table 1). The emergence of *C. haematopotus* was significantly higher from the stream sites than from puddle and seepage sites (LR $\chi^{ 2}_{\left( 2\right)}$ = 72.4, *P* < 0.0001). In the puddle habitats, *C. stellifer* was the most abundant species (49.5%, 94/190) followed by *C. haematopotus* (36.3%, 69/190). The emergence of *C. stellifer* was significantly higher from the puddle edge sites than from stream edge or seepage sites (LR $\chi^{ 2}_{\left( 2\right)}$ = 68.7, *P* < 0.0001). The emergence of *C. loisae* was significantly higher from the stream sites than from the puddle or seepage sites (LR $\chi^{ 2}_{\left( 2\right)}$ = 9.7, *P* = 0.0078). *Culicoides venustus* emerged only from the puddle and seepage sites but not from stream sites (Table 1). The differences in the emergence of *C. venustus* between the two habitats were marginally significant; however, the Tukeyʼs test did not detect significant differences between individual habitat types (LR $\chi^{ 2}_{\left( 1\right)}$ = 4.0, *P* = 0.0454). Overall, *C. haematopotus* (79.8%, 280/351) and *C. loisae* (70.2%, 33/47) emerged primarily from the stream edge sites, while *C. stellifer* emerged mainly from the puddle edge habitats (81.0%, 94/116) (Table 1). *Culicoides venustus* did not show associations with any particular habitat type but appeared to avoid the stream edge sites.

The overall sex-ratio of the emerged adults was slightly female biased in *C. haematopotus* [~ 1:2 (male:female)], but was ~ 1:1 in *C. stellifer*, *C. loisae* and *C. venustus* (Table 1). However, this variation in sex-ratios was not found to be significantly different between species (LR $\chi^{ 2}_{\left( 3\right)}$ = 2.3, *P* = 0.5165) and habitat types (LR $\chi^{ 2}_{\left( 2\right)}$ = 0.4, *P* = 0.8055), nor was the interaction effect significant (LR $\chi^{ 2}_{\left( 2\right)}$ = 0.4, *P* = 0.8238).

**Fig. 2:**
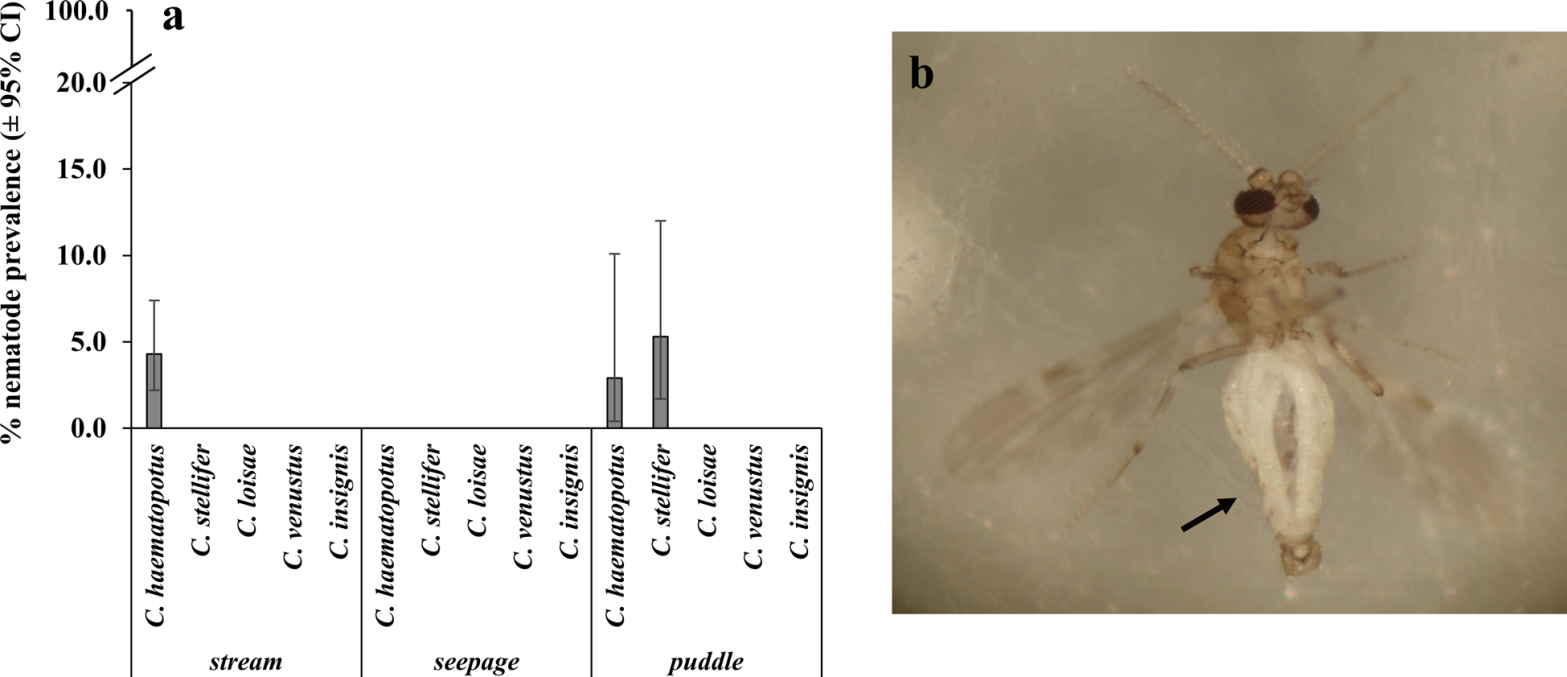
**a** Percentage prevalence (mean ± 95% CI) of parasitic nematodes in the emerged adults by species and habitat type. **b** An adult midge parasitized by nematodes. Notice the male terminalia and female type antennae in the individual shown (intersex). Arrow points to the midge abdomen where nematodes can be seen as translucent coils. The prevalence of parasitic nematodes was not significantly different between *Culicoides* species and habitat types (*P* > 0.05)

A small percentage of the emerged adults were found to be parasitized by nematodes, which were assumed to be members of the family Mermithidae (see Discussion section for our justification). Only two species, *C. haematopotus* (overall prevalence 4.0%; 95% CI: 2.2–6.6%) and *C. stellifer* (4.3%; 95% CI: 1.4–9.8%) were parasitized, but not the other species across all three sampling months (Fig. 2a). Moreover, nematodes were found only in the adults that emerged from stream (3.7%; 95% CI: 1.9–6.4%) and puddle sites (3.7%; 95% CI: 1.5–7.4) but not from those that emerged from seepage sites (Fig. 2a). However, the prevalence of parasites in the adults was not found to be significantly different between species (LR $\chi^{ 2}_{\left( 4\right)}$ = 5.1, *P* = 0.2819) and habitat types (LR $\chi^{ 2}_{\left( 2\right)}$ = 1.3, *P* = 0.5185), with no significant interaction effect either (LR $\chi^{ 2}_{\left( 6\right)}$ = 1.3, *P* = 0.9715).

### Variation in environmental variables between habitat types

Stream sites, in general, had the lowest concentrations/levels of almost all environmental variables quantified; seepage sites had intermediate concentrations/levels, while puddle sites had the highest (Table 2). The concentrations/levels of copper (Cu), pH and microbes were not significantly different between the three habitat types (Table 2).

**Table 2:** Mean concentrations/levels (± SE) of different environmental variables quantified from the three habitat types

Environmental variable	Units	Stream	Seepage	Puddle	*F*-statistic (Num*df*, Den*df*^a^)	*P-*value^b^
Organic matter	%	1.5 ± 0.3 a	4.3 ± 1.3 a	16.6 ± 3.2 b	11.7 (2, 3)	**0.0381**
Phosphorus	mg/kg	39.8 ± 4.8 b	22.8 ± 2.9 a	111.1 ± 6.8 c	45.7 (2, 3)	**0.0057**
Potassium	mg/kg	10.1 ± 1.7 a	26.7 ± 3.7 b	66.5 ± 4.4 c	29.8 (2, 3)	**0.0105**
Magnesium	mg/kg	22.1 ± 4.9 a	87.4 ± 11.1 b	144.2 ± 12.0 b	30.4 (2, 3)	**0.0102**
Calcium	mg/kg	278.2 ± 52.2 a	334.3 ± 32.0 a	1059.1 ± 63.2 b	29.5 (2, 3)	**0.0106**
Copper	mg/kg	0.0 ± 0.0 a	0.0 ± 0.0 a	0.03 ± 0.01 a	7.7 (2, 3)	0.0661
Manganese	mg/kg	2.4 ± 0.9 a	6.7 ± 0.9 b	19.3 ± 2.5 c	32.6 (2, 3)	**0.0092**
Zinc	mg/kg	0.4 ± 0.1 a	0.9 ± 0.1 b	3.0 ± 0.2 c	48.4 (2, 3)	**0.0052**
pH	pH	5.3 ± 0.1 a	5.0 ± 0.1 a	5.2 ± 0.1 a	0.7 (2, 3)	0.5683
Electrical conductivity	dS/m	0.1 ± 0.0 a	0.2 ± 0.0 b	0.3 ± 0.0 c	13.0 (2, 3)	**0.0334**
Moisture	%	34.2 ± 2.9 a	59.5 ± 2.8 b	70.1 ± 1.8 c	59.0 (2, 3)	**0.0039**
Microbes	CFU/ml	1.1 ± 0.2 × 10^8^ a	9.1 ± 2.2 × 10^7^ a	5.1 ± 1.0 × 10^8^ a	4.0 (2, 3)	0.1412

^a^Denominator degrees of freedom (Den*df*) were estimated using the Kenward–Roger method

^b^Significant *P-*values are shown in bold

*Note*: Means followed by different letters indicate significant differences between habitat types (*P* < 0.05)

### Influence of environmental variables on *Culicoides* emergence

The number of best AICc ranked models that were selected to calculate the predictive averaged models (delta AICc < 2) for *C. haematopotus*, *C. loisae*, *C. stellifer* and *C. venustus* were 2 (out of 256 total models generated), 8 (out of 256 models), 7 (out of 2048 models) and 14 (out of 2048 models), respectively. The coefficients and Akaike’s weights estimated using the averaged models revealed that the relative importance of environmental variables within the most productive habitat(s) in determining midge abundance varied between the four *Culicoides* species. The abundance of *C. haematopotus* within the stream habitat was mainly positively associated with moisture, phosphorus, pH, zinc and microbial concentrations in the substrate, but was negatively related to electrical conductivity and manganese levels (Table 3). On the other hand, the abundance of *C. loisae* within the stream habitat was positively associated with phosphorus, organic matter and zinc levels in the substrate but was negatively related to pH, electrical conductivity and manganese levels (Table 4). The abundance of *C. stellifer* within the puddle habitat was mainly positively associated with potassium and zinc levels, but was negatively related to pH, phosphorus, electrical conductivity, magnesium and organic matter levels of the substrate (Table 5). Finally, the abundance of *C. venustus* across both the habitats this species emerged from (puddles and seepages) was mainly positively related to microbes, zinc, phosphorus, magnesium and electrical conductivity levels of the substrate but was negatively associated with organic matter, manganese and copper levels (Table 6). However, as evident from the ‘estimates’, only weak associations were detected between the abundance of each *Culicoides* species and the various environmental variables quantified (Tables 3–6).

**Table 3:** Model averaging summary showing the magnitude of effect and relative importance of different environmental variables in stream habitats on the abundance of *C. haematopotus*

Environmental variable	Estimate (SE)	Relative variable importance	*n* (containing models)
Intercept	1.66 (0.52)		
Electrical conductivity	− 1.84 (0.34)	1.00	2
Manganese	− 0.85 (0.24)	1.00	2
Moisture	0.77 (0.21)	1.00	2
Phosphorus	1.01 (0.21)	1.00	2
pH	0.55 (0.10)	1.00	2
Zinc	1.02 (0.29)	1.00	2
Microbes	0.25 (0.20)	0.71	1

*Abbreviation*: SE, standard error

**Table 4:** Model averaging summary showing the magnitude of effect and relative importance of different environmental variables in stream habitats on the abundance of *C. loisae*

Environmental variable	Estimate (SE)	Relative variable importance	*n* (containing models)
Intercept	− 0.63 (0.48)		
Phosphorus	0.75 (0.62)	0.71	6
pH	− 0.56 (0.52)	0.67	5
Electrical conductivity	− 0.84 (0.98)	0.53	4
Manganese	− 0.52 (0.91)	0.34	3
Organic matter	0.23 (0.47)	0.26	2
Zinc	0.07 (0.28)	0.09	1

*Abbreviation*: SE, standard error

**Table 5:** Model averaging summary showing the magnitude of effect and relative importance of different environmental variables in puddle habitats on the abundance of *C. stellifer*

Environmental variable	Estimate (SE)	Relative variable importance	*n* (containing models)
Intercept	0.76 (0.49)		
pH	− 0.31 (0.25)	0.67	4
Phosphorus	− 0.35 (0.35)	0.66	5
Electrical conductivity	− 0.12 (0.20)	0.35	2
Potassium	0.22 (0.34)	0.33	3
Magnesium	− 0.17 (0.29)	0.33	3
Organic matter	− 0.10 (0.21)	0.24	2
Zinc	0.04 (0.14)	0.11	1

*Abbreviation*: SE, standard error

**Table 6:** Model averaging summary showing the magnitude of effect and relative importance of different environmental variables across puddle and seepage habitats on the abundance of *C. venustus*

Environmental variable	Estimate (SE)	Relative variable importance	*n* (containing models)
Intercept	− 1.51 (0.40)		
Organic matter	− 1.51 (0.79)	1.00	14
Microbes	0.25 (0.22)	0.72	10
Zinc	0.51 (0.54)	0.59	9
Phosphorus	0.31 (0.33)	0.58	8
Magnesium	0.14 (0.22)	0.39	5
Manganese	− 0.11 (0.25)	0.21	3
Copper	− 0.05 (0.15)	0.13	2
Electrical conductivity	0.02 (0.09)	0.05	1

*Abbreviation*: SE, standard error

## Discussion

Overall, our findings demonstrate that habitat associations of *Culicoides* species occurring/abundant on a local cervid/animal farm vary, most likely as a function of certain biotic/abiotic characteristics of the habitat. Among the five *Culicoides* species that emerged across the samples, distinct habitat associations were observed only for *C. haematopotus* and *C. loisae* that emerged primarily from the stream edge sites, and *C. stellifer* that emerged mainly from the puddle sites, suggesting that these sites are more favorable for the oviposition and/or larval development of these respective midge species. *Culicoides venustus*, although not showing any distinct habitat associations, emerged only from puddle and seepage sites but not from the stream sites. On the other hand, the emergence of *C. insignis* was too low to form any meaningful conclusions. In general, our findings are consistent with previous reports of *C. haematopotus* and *C. loisae* emerging mainly from the margins of streams and ponds where pollution is usually low [28]. Similarly, our findings are also consistent with previous reports on *C. stellifer* larvae occurring mainly in shaded muddy areas with leaf litter/organic debris [28]. However, in the present study, *C. venustus* was not associated with any particular habitat but appeared to avoid the stream sites. Interestingly, previous studies reported *C. venustus* to be associated with wet pastures developing mainly in muddy hoofprints of livestock [28]. Thus, our findings of *C. venustus* emerging from puddles and seepages extends the known habitats of this species, suggesting that *C. venustus* may occupy more diverse habitats than previously thought. However, it is important to note that the five *Culicoides* species that emerged from our samples may not represent the overall adult *Culicoides* diversity on this property because midge emergence from other types of habitats such as tree holes and others were not examined in the present study. Previously, light trap collections and/or aspirations from white-tailed deer on this property revealed *C. stellifer*, *C. haematopotus*, *C. venustus*, *C. debilipalpis* and/or *Culicoides pallidicornis* (Kieffer) to be the dominant midge species at this site [32, 53]. However, the abundance of different *Culicoides* species varied with season and also with the height of collection [32, 53].

Among the five *Culicoides* species that emerged across our samples, *C. loisae* is considered non-hematophagous owing to the absence of mandibular teeth and reduced tormae [28], and thus, is not likely to be involved in BTV/EHDV transmission. However, the other four *Culicoides* species have all been associated with BTV/EHDV transmission in North America in the past. *Culicoides haematopotus* is found throughout the USA usually in wooded areas and the larvae frequently occur along the margins of streams and ponds that are not usually heavily polluted [28]. This species is a generalist feeder and has been collected from cattle in Alabama [54] and deer in Georgia at a site enzootic for hemorrhagic disease [17], and field-collected individuals from Louisiana were found to be positive for BTV [55]. However, a recent study from Florida study suggested that *C. haematopotus* may not play a major role in BTV/EHDV transmission in this site/region [32]. *Culicoides stellifer* occurs throughout most of the USA usually around livestock and the larvae occur in a variety of organically enriched habitats [28]. This species seeks blood meals in large numbers from a variety of mammals including white-tailed deer and cattle [14, 16, 17, 32, 54, 56, 57], and field-collected individuals from Florida were found to be positive for EHDV [58], making it a strong suspected vector of BTV/EHDV, particularly in southeastern USA. *Culicoides venustus* is found mainly in eastern USA and the larvae usually occur in wet pastures often in the muddy hoof prints of livestock [28]. This species is nocturnal and has been reported to bite livestock [56, 57, 59–61], and field-collected individuals from Alabama and Florida were found to be positive for EHDV [58], making it another strong suspected vector of BTV/EHDV in southeastern USA. Interestingly, vector competence studies on *C. venustus* suggested the New York population to be an inefficient vector of BTV/EHDV [62]. However, whether *C. venustus* populations from Florida and other neighboring states are competent vectors of BTV/EHDV remains to be examined in further studies. *Culicoides insignis* is a confirmed vector of BTV in North America [9] and a likely vector of EHDV; however, studies incriminating this species for EHDV transmission are lacking. The distribution of *C. insignis* in the USA is currently limited to a few southeastern states, but recent findings indicate a northward range expansion in this species [10]. *Culicoides insignis* is often associated with livestock operations and the larvae occur mainly in muddy areas in cow pastures or along the margins of vegetated ponds, but also in other habitats [28, 63–65]. This species seeks blood meals from livestock in large numbers that, apart from resulting in pathogen transmission, can also induce hypersensitivity reactions in susceptible hosts [60]. Nonetheless, *C. insignis* emergence in our study was very low (*n* = 2 females), which is consistent with previous reports of this species being rare at this site [32]. Therefore, *C. insignis* may not play a major role in BTV/EHDV transmission in this region. Nevertheless, the vector competence of all these hematophagous *Culicoides* species (particularly the populations from southeastern USA) for BTV and/or EHDV is currently unknown and remains to be examined in further studies.

Parasitization of ceratopogonids by mermithid nematodes has been reported worldwide including in certain *Culicoides* species in North America [66]. In the present study, a small percentage of *C. haematopotus* and *C. stellifer* midges were parasitized by nematodes, which were assumed to be mermithids. Our assumption that these nematodes were mermithids was based on (i) our observations that the parasitized *Culicoides* adults were intersexes, i.e. individuals with male genitalia but with female type antennae (Fig. 2b); (ii) previous reports of *C. haematopotus* and *C. stellifer* being parasitized by mermithid nematodes in southeastern USA [34–36]; and (iii) previous reports of intersex *Culicoides* adults resulting from parasitism by mermithid nematodes [34–36]. Interestingly, although not statistically significant, only *C. haematopotus* and *C. stellifer* were found to be parasitized by nematodes, but not the other species across the study. Moreover, only midges that emerged from the stream and puddle sites (sites that *C. haematopotus* and *C. stellifer* showed associations for) were parasitized, but not the midges that emerged from seepage sites. This suggests a possible exhibition of (i) host specificity by the nematodes for the two *Culicoides* species, and/or (ii) site specificity by the nematodes for the habitats that these midge species develop and/or are more abundant in. Previously, the mermithid nematode *Heleidomermis magnapapula* Poinar & Mullens was found to exhibit high host specificity to *C. sonorensis* and also high site specificity to the manure-enhanced mud habitats of *C. sonorensis* [67]. Furthermore, although parasitization rates were low in *C. sonorensis* adults, these were found to be much higher in the larvae [67]. Moreover, preliminary field release trials of this nematode showed promising results for the control of *C. sonorensis* in California [66]. Currently, very little is known regarding the dynamics/extent of nematode parasitization in *Culicoides* species in southeastern USA [34–36]. Further studies will be needed to (i) identify the nematode species parasitizing *C. haematopotus* and *C. stellifer*; (ii) examine the patterns of parasitism including host specificity, site specificity, parasitism rates in different midge life stages and distribution; and (iii) evaluate whether these nematode species can be cultured and exploited as biocontrol agents against target *Culicoides* species in southeastern USA.

Most of the environmental variables in stream edge sites were at concentrations/levels much lower than those in the seepage and puddle sites. This is not unexpected, because flowing water of the stream likely prevents accumulation of organic matter and/or other biotic/abiotic factors within and flushes out the incoming nutrients. The other habitats, in contrast, particularly the puddle sites, had the highest concentrations/levels of almost all the environmental variables measured, which can be attributed to stagnant water conditions and accumulation of various biological and physicochemical factors within these sites. The sources from which puddle sites receive heavy inflows of biotic/abiotic factors are currently unknown. However, puddle sites likely receive large amounts of nutrients when the stream rises and floods the low-lying areas after major storms and when nutrients from higher fields are washed downhill into the lowland puddles. Additionally, these data suggest a frequent deposition of animal manure/urine in the puddle sites. Ruminant manure/urine, in general, contains high concentrations/levels of nutrients such as phosphorus, potassium, magnesium, calcium and nitrogen, among others, and can alter the soil chemistry of the habitats [68–72]. As such, many of the environmental variables of the substrates measured in this study can serve as indicators of animal access to the site and thus indicate pollution levels. Indeed, animals on the property were found to frequently visit the puddle sites and leave visible animal hoof prints and manure pats in these habitats. Thus, the puddle sites contained overall higher pollution levels than in the other sites examined. The seepage sites had more visible leaf litter/debris than the other two habitats and animals were noticed in these areas on occasion. This potentially explains the intermediate concentrations/levels of some environmental variables in the seepage sites, thus indicating moderate pollution levels in these seepage habitats.

The present study demonstrates that several ground-dwelling *Culicoides* species other than *C. sonorensis* occur abundantly on a local cervid farm in Florida occupying a variety of habitats, most likely as a function of certain biotic/abiotic factors in the habitat. The hematophagous *Culicoides* species that was more abundant in our samples, *C. haematopotus*, was associated with habitats with low pollution levels such as the edges of streams while *C. stellifer* was associated with habitats having higher pollution levels such as the edges of puddles. On the other hand, although *C. venustus* did not show any distinct habitat associations, it emerged only from the puddle and seepage sites that had relatively higher pollution levels, but not from the stream sites that had low pollution levels, suggesting that this species develops primarily in habitats with moderate-high pollution levels (Table 1). Nonetheless, as evident from the ‘estimates’, only weak associations were detected between *Culicoides* species abundance and the various biotic/abiotic factors examined with the environmental variables positively or negatively influencing midge abundance and their relative importance varying between *Culicoides* species (Tables 3, 4, 5, 6). Therefore, further studies will be needed across a larger spatial and temporal scale (along with screening for more biotic/abiotic factors) to identify variables that are more strongly associated with the abundance of target *Culicoides* species. Interestingly, what stands out in these analyses, particularly for *C. stellifer*, is that although this species emerged mainly from the puddle sites that had the highest concentrations/levels of almost all the environmental variables measured including organic matter, negative associations were detected between *C. stellifer* abundance and organic matter (Table 5). This seems counterintuitive because *C. stellifer* has been previously reported to occur in muds enriched with various types of organic matter. Similarly, although *C. venustus* generally emerged mainly from habitats with moderate-high levels of pollution (puddles and seepages), its abundance was negatively associated with organic matter (Table 6). However, previous studies reported *C. venustus* to be mainly associated with wet pastures with the larvae occurring in hoof prints of livestock [28]. Therefore, we hypothesize that extremely high concentrations/levels of organic matter in the substrate may not be ideal for *C. stellifer* and *C. venustus* oviposition/larval development, but a low-moderate amount of organic matter may be required for the occurrence of these species at a potential site. Previously, certain members of *C. variipennis* complex were found to show positive correlations to the degree of animal access to a potential site [73, 74]; however, concentrations of organic matter in the substrate were found to be negatively correlated to their abundance [74]. Subsequent studies showed that habitats with intermediate levels of manure pollution (500–1500 mg COD/liter) were more suitable for *C. sonorensis* than those with lower or higher manure pollution levels [24]. More recently, *C. sonorensis* larval development was found to be optimal when manure concentration in the substrate was 25.0%, but decreased at lower or higher manure concentrations [19]. However, further studies will be needed to test these hypotheses in *C. stellifer* and *C. venustus*.

Currently, very little is known regarding the habitat requirements of *C. haematopotus*, *C. stellifer* and *C. venustus* [28]. A recent study examined the oviposition stimuli of *C. stellifer* under laboratory conditions and suggested that muds enhanced with white-tailed deer manure may not be particularly attractive for oviposition in this species [31]. This is intriguing because *C. stellifer* in the present study emerged mainly from puddle sites that had the highest pollution levels among the three habitats. It is possible that the puddle sites may be polluted with cervid as well as bovid manure or may be even polluted more with bovid manure than cervid manure, which could be due to the differential frequency of animal visits to these sites on the property (not noted in this study). Previously, different animal manures were found to be differentially attractive for oviposition in *Culicoides* species and other insects [75–77]. Moreover, bovid manure was shown to be better than cervid manure in supporting the larval development of *C. sonorensis* [19]. Alternately, certain types of vegetation (not noted in this study) could be more closely associated with certain habitats owing to differences in soil chemistry profiles across the landscape (as shown in this study between different habitats) and play a role in the habitat site selection of *Culicoides* species. Previously, vegetation (*Sphagnum* spp. mosses) from the midge habitat was found to elicit a strong oviposition response under laboratory conditions in *C. stellifer* [31] and other species [78], while several *Culicoides* species were found to be spatially associated with certain types of vegetation in the field [28, 63, 79, 80]. It is also possible that other environmental variables that were not examined in this study could be more important for *Culicoides* species occurrence/abundance at a potential site. Nonetheless, much of the information on habitat characteristics of *C. stellifer* and other *Culicoides* species is currently unknown. Further studies will be needed to examine (i) whether bovid manure is more attractive for midge oviposition than cervid manure; (ii) whether bovid manure is better than cervid manure in supporting the larval development of *Culicoides* species more abundant in southeastern USA; (iii) whether cervids and bovids differ in their spatial use of a landscape; and (iv) whether certain types of vegetation (or other unexamined environmental variables) are more closely associated with certain habitats and play a role in the habitat site selection of *Culicoides* species. Further studies will also be needed to examine other aspects of the biology/ecology of *C. haematopotus*, *C. stellifer* and *C. venustus* as well as their vector competence for BTV/EHDV as very little is known about these species and their role in the transmission of orbiviruses in North America, particularly in southeastern USA.

## Conclusions

Our study demonstrates that the habitat associations of ground-dwelling *Culicoides* species abundant on a local cervid/animal farm vary, most likely as a function of certain biotic/abiotic characteristics of the habitat. More specifically, *C. haematopotus* and *C. loisae* emerge mainly from sites with low eutrophic conditions such as the edges of streams while *C. stellifer* and *C. venustus* emerge mainly from sites with moderate-heavy eutrophic conditions such as the edges of puddles and seepages. The habitat associations of *C. insignis* currently remain uncertain. Overall, our study provides valuable information regarding the habitat associations of *Culicoides* species that are abundant on a commercial cervid farm in Florida, and moreover are suspected vectors of Orbiviruses in southeastern USA. Furthermore, to our knowledge, our study provides the first insight into some of the key environmental variables potentially important in the abundance of these understudied midge species. However, further studies on a larger spatial and temporal scale will be needed to experimentally evaluate and identify the key environmental variables that are more strongly associated with midge abundance. This information, in the long term, can be potentially exploited to render local habitats unsuitable for midge oviposition/larval development.

## Additional file

**Additional file 1: Table S1.** Collinearities in the environmental variables within stream habitat. The initial full model is represented by environmental variables listed in column VIF1. Columns VIF2 and VIF3 represent removal of collinearity by sequentially deleting the variable with the highest VIF value. Column VIF4 represents the final model containing environmental variables with only VIF values < 10. **Table S2.** Collinearities in the environmental variables within puddle habitat. The initial full model is represented by environmental variables listed in column VIF1. Column VIF2 represents the final model containing environmental variables with only VIF values < 10. **Table S3.** Collinearities in the environmental variables across puddle and seepage habitats. The initial full model is represented by environmental variables listed in column VIF1. Column VIF2 represents the final model containing environmental variables with only VIF values < 10.

## Data Availability

All data generated/analyzed during the study are included in this article and its additional file.

## Abbreviations

BTV: bluetongue virus
EHDV: epizootic hemorrhagic disease virus
CI: confidence interval
L:D: light:dark
RH: relative humidity
