# Supplementary material for: Habitat associations of Culicoides species (Diptera: Ceratopogonidae) abundant on a commercial cervid farm in Florida, USA
Source: Parasit Vectors. 2019 Jul 26;12:367. doi: 10.1186/s13071-019-3626-1 (PMC6660662; doi:10.1186/s13071-019-3626-1)
Supplement: Supplementary file 1 — Additional file 1: Table S1. Collinearities in the environmental variables within stream habitat. The initial full model is represented by environmental variables listed in column VIF1. Columns VIF2 and VIF3 represent removal of collinearity by sequentially deleting the variable with the highest VIF value. Column VIF4 represents the final model containing environmental variables with only VIF values < 10. Table S2. Collinearities in the environmental variables within puddle habitat. The initial full model is represented by environmental variables listed in column VIF1. Column VIF2 represents the final model containing environmental variables with only VIF values < 10. Table S3. Collinearities in the environmental variables across puddle and seepage habitats. The initial full model is represented by environmental variables listed in column VIF1. Column VIF2 represents the final model containing environmental variables with only VIF values < 10. [file 13071_2019_3626_MOESM1_ESM.docx]

**Additional file 1**

**Table S1.** Collinearities in the environmental variables within stream habitat. The initial full model is represented by environmental variables listed in column VIF1. Columns VIF2 and VIF3 represent removal of collinearity by sequentially deleting the variable with the highest VIF value. Column VIF4 represents the final model containing environmental variables with only VIF values < 10.

| **Environmental variable** | **VIF1** | **VIF2** | **VIF3** | **VIF4** |
| --- | --- | --- | --- | --- |
| Microbes | 3.1 | 2.6 | 2.4 | 2.4 |
| pH | 3.3 | 3.3 | 1.9 | 1.7 |
| Copper | NA | NA | NA | NA |
| Moisture | 7.8 | 7.3 | 6.6 | 5.8 |
| Manganese | 19.6 | 13.2 | 10.4 | 7.8 |
| Electrical conductivity | 14.5 | 13.6 | 11.7 | 9.8 |
| Magnesium | 63.0 | NA | NA | NA |
| Phosphorus | 27.6 | 21.3 | 21.2 | 7.0 |
| Zinc | 13.7 | 7.6 | 7.6 | 6.1 |
| Potassium | 39.9 | 31.8 | 26.1 | NA |
| Calcium | 39.4 | 35.3 | NA | NA |
| Organic matter | 9.8 | 9.7 | 9.7 | 7.6 |

NA indicates that the variable was removed due to concentrations/levels being zero (copper) or due to high VIF value (VIF2 & VIF3).

**Table S2.** Collinearities in the environmental variables within puddle habitat. The initial full model is represented by environmental variables listed in column VIF1. Column VIF2 represents the final model containing environmental variables with only VIF values < 10.

| **Environmental variable** | **VIF1** | **VIF2** |
| --- | --- | --- |
| Microbes | 1.9 | 1.8 |
| pH | 1.8 | 1.7 |
| Copper | 3.8 | 3.8 |
| Moisture | 3.4 | 3.2 |
| Manganese | 3.9 | 3.8 |
| Electrical conductivity | 2.5 | 2.3 |
| Magnesium | 2.8 | 2.6 |
| Phosphorus | 3.0 | 2.4 |
| Zinc | 9.0 | 5.7 |
| Potassium | 4.1 | 2.4 |
| Calcium | 10.4 | NA |
| Organic matter | 4.3 | 3.4 |

NA indicates that the variable was removed due to high VIF value.

**Table S3.** Collinearities in the environmental variables across puddle and seepage habitats. The initial full model is represented by environmental variables listed in column VIF1. Column VIF2 represents the final model containing environmental variables with only VIF values < 10.

| **Environmental variable** | **VIF1** | **VIF2** |
| --- | --- | --- |
| Microbes | 1.6 | 1.6 |
| pH | 1.5 | 1.5 |
| Copper | 3.2 | 3.2 |
| Moisture | 2.3 | 2.3 |
| Manganese | 2.6 | 4.3 |
| Electrical conductivity | 2.2 | 2.1 |
| Magnesium | 4.4 | 2.5 |
| Phosphorus | 5.0 | 4.9 |
| Zinc | 9.1 | 7.2 |
| Potassium | 3.8 | 3.3 |
| Calcium | 10.4 | NA |
| Organic matter | 3.2 | 2.4 |

NA indicates that the variable was removed due to high VIF value.
